# Semivariogram and Semimadogram functions as descriptors for AMD diagnosis on SD-OCT topographic maps using Support Vector Machine

**DOI:** 10.1186/s12938-018-0592-3

**Published:** 2018-10-23

**Authors:** Alex M. Santos, Anselmo C. Paiva, Adriana P. M. Santos, Steve A. T. Mpinda, Daniel L. Gomes, Aristófanes C. Silva, Geraldo Braz, João Dallyson S. de Almeida, Marelo Gattass

**Affiliations:** 1 Federal University of Maranhão UFMA, Applied Computing Group - NCA, Av. dos Portugueses, SN, Campus do Bacanga, Bacanga, São Luís, MA 65085-580 Brazil; 2 Instituto Federal de Educação, Ciência e Tecnologia do Maranhão, São José de Ribamar, MA Brazil; 3 Université du Québec à Montréal, Montréal, Canada; 4 Pontifical Catholic University of Rio de Janeiro PUC-Rio, R. São Vicente, 225 Gávea, Rio de Janeiro, RJ 22453-900 Brazil

**Keywords:** Medical images, Optical coherence tomography, CAD-x, Semivariogram, Semimadogram

## Abstract

**Background:**

Age-related macular degeneration (AMD) is a degenerative ocular disease that develops by the formation of drusen in the macula region leading to blindness. This condition can be detected automatically by automated image processing techniques applied in spectral domain optical coherence tomography (SD-OCT) volumes. The most common approach is the individualized analysis of each slice (B-Scan) of the SD-OCT volumes. However, it ends up losing the correlation between pixels of neighboring slices. The retina representation by topographic maps reveals the similarity of these structures with geographic relief maps, which can be represented by geostatistical descriptors. In this paper, we present a methodology based on geostatistical functions for the automatic diagnosis of AMD in SD-OCT.

**Methods:**

The proposed methodology is based on the construction of a topographic map of the macular region. Over the topographic map, we compute geostatistical features using semivariogram and semimadogram functions as texture descriptors. The extracted descriptors are then used as input for a Support Vector Machine classifier.

**Results:**

For training of the classifier and tests, a database composed of 384 OCT exams (269 volumes of eyes exhibiting AMD and 115 control volumes) with layers segmented and validated by specialists were used. The best classification model, validated with cross-validation k-fold, achieved an accuracy of 95.2% and an AUROC of 0.989.

**Conclusion:**

The presented methodology exclusively uses geostatistical descriptors for the diagnosis of AMD in SD-OCT images of the macular region. The results are promising and the methodology is competitive considering previous results published in literature.

## Background

Age-related macular degeneration (AMD) is a progressive chronic disease of the central retina and a leading cause of vision loss worldwide [1]. Its development is characterized by the appearance of soft drusen (discrete deposits of residues) and abnormalities in pigmentation of the retinal sublayer known as retinal pigment epithelium (RPE) [2]. Damage in this region causes the inability to read, to recognize faces, etc. Its progression can lead to vision impairment, or even total blindness.

It is estimated that AMD is the cause of 8.7% of cases of blindness worldwide, with a higher incidence in developed countries and people over 60 years of age. For 2020, the global projected number of cases is of 196 million and this estimation reaches 288 million for the year 2040 [3].

Recent advances in retinal imaging technology have allowed the handling of age-related macular degeneration through the creation of spectral domain optical coherence tomography (SD-OCT). It is a noncontact, noninvasive, three-dimensional imaging technique that obtains images representing a sectional view of retina at micrometer resolution [4].

This examination modality reconstructs the tomographic image by measuring the optical backscattering of tissues. This technique has gained space as a diagnostic tool for AMD, mainly due to its quickness to acquire images and dispenses the use of contrasts and thus the risks involved.

The evaluation of AMD by SD-OCT occurs through the analysis of successive sectional cuts in search of morphological changes caused by the disease. SD-OCT acquisition generates a large number of sectional images, which requires that the expert visualize and evaluate a large number of volume slices seeking for changes, especially if they are minimal and sparse in the early stages of pathology [5].

A typical SD-OCT examination produces about 100 images with a resolution of $512 \times 1000$ pixels (fast acquisition protocol). This demands considerable human effort for evaluation. If we consider that a physician is responsible for the care and follow-up of dozens of patients, there is a necessity for technologies that support the clinical practice of SD-OCT image analysis.

Accordingly, the development of computer aided diagnostic methods (CADx) can collaborate with the specialist for the detection of AMD by taking advantage of the ability to evaluate the large amount of data that the SD-OCT examination can provide. Even in its variations, a large amount of anatomical retinal information is available to characterize the disease [6].

Typically, we have two main approaches for automated AMD diagnosis based on SD-OCT. One of the approaches involves the following steps to be effectively addressed: (a) obtaining relevant biomedical characteristics for the differentiation between healthy and compromised retinas; (b) use of classifiers to accurately determine the presence or absence of the disease; and (c) validation of the method for generating reliable results from a properly classified image database [4, 5, 7–13]. This approach has the advantage of constructing robust classifiers, based on fixed mathematical models that can characterize well a specific pathology.

Another approach is based on the use of deep neural networks techniques [14] to identify pathologies that compromise the retina [15–19]. These methods present the main advantage of flexibility for training to discriminate various conditions, especially AMD and diabetic macular edema, and also do not require the search for discriminatory characteristics which are determined by the deep learning framework.

In general, these methods use the reflectance values of retinal pixels. They use simple segmentation techniques based on the elimination of the background [8], a warping of the image from flattening the retina [4, 13, 17], a cut of predefined dimensions in the region of greatest intensity of pixels [9] or even semi-automatic segmentation of retinal layers [5] to delimit the region of interest.

The main related works are compared in Table 1. We can notice that in some works the images are pre-processed for the normalization of intensity values of pixels [7] and noise reduction. This filtering is normally done with filters based on wavelet [20] or with the Total Variation algorithm [8, 10]. The main advantage of these techniques is providing better definition of anatomic alterations that distinguish images compromised by AMD.

**Table 1:** Related work comparison

Work	Image representation	Preprocessing	Features	Classifier	Volumes	Images are publicly available
Liu et al. [4]	2D	Image warping	Multi-scale spatial pyramid, LBP histogram + PCA	Non-linear Support Vector Machine	457	No
Serrano et al. [7]	2D	Normalization	Haar-Like features and Haralick texture features (curtosis and skewness)	Decision Trees	200	No
Albarrak et al. [8]	3D	Split Bregman Isotropic Total Variation algorithm and a second order polynomial least-square curve fitting for image flattening	Oriented gradient local binary pattern histograms	Bayes network	140	No
Zhang et al. [10]	3D	Bregman Isotropic Total Variation algorithm with a least squares approach	Local binary patterns of three orthogonal planes (LBP-TOP), local phase quantization (LPQ) and multi-scale spatial pyramid (MSSP)	Ensemble of one-class kernel principal component analysis (KPCA) models	140	No
Farsiu et al. [5]	3D	Segmentation of tree retinal layers	Abnormal RPEDC thickness and thinness scores	Generalized linear model regression	384	Yes
Srinivasan et al. [9]	3D	Denoise with BM3D	HOG descriptors	Three linear one-class Support Vector Machines	45	Yes
Venhuizen et al. [11]	2D	First order vertical Gaussian gradient filter	Unsupervised feature learning approach based in patches of images	Random forest classifier	384	Yes
Wang et al. [12]	2D	–	Multi-scale linear configuration patterns (LCP)	Sequential minimal optimization (SMO)	45	Yes
Sun et al. [13]	2D	Retina aligning and crop SIFT descriptors		Three two-class Support Vector Machines (SVM)	45/678 scans	Yes/no
Ravenscroft et al. [15]	2D	Manual segmentation and labelling of choroid	Learnable features by Convolutional Neural Network (CNN)	Neural Network	75	No
Fang et al. [16]	3D	Patch mean removal	PCA features	Extreme learning machine (ELM) classifier	45/54	Yes/no
Karri et al. [17]	2D	RPE estimation based in intensity and BM3D filter is used for noise reduction	Learnable features	Convolutional Neural Network (Transfer learning/GoogLeNet)	45	Yes
Lee et al. [18]	2D	–	Learnable features	Convolutional Neural Network	100,000 B-scans	No
Kermany et al. [19]	2D	–	Learnable features	Convolutional Neural Network (Transfer Learning)	207,130 B-scans	Yes

Most described methods are based on the individual analysis of the SD-OCT volumes’ B-Scans, not considering the correlation between successive slices as seen in [12, 13, 15, 21]. This type of application is common mainly due to being more accessible to define a region of interest from a B-Scan than from any other volume representation. Normally, the successive slices suffer a misalignment caused by eye movement during capture. Thus, it becomes more practical that each slice be processed individually.

The main disadvantage of methods that analyze individual B-scans is to disregard the correlation between pixels of successive slices. Moreover, after the step of detecting characteristics that indicate the presence of AMD per slice, a new classification is necessary to indicate the presence of the disease by volume.

The approach presented in [5] differs from others by representing the SD-OCT volume in thickness maps of total retina (TR), neurosensory retina (NSR), and especially of the retinal pigmented epithelium and drusen complex (RPEDC), which region has a higher incidence of AMD-induced alterations. With this, it is possible to generate a representation of a whole SD-OCT volume for the detection of AMD. Following, the topographic maps generated are redimensioned from $1000 \times 100$ to $1001 \times 1001$ pixels, reconstructing the original appearance of the retina, however at the expense of inserting interpolated data in the image.

From this point, they generate mean thickness maps and determine superior and inferior limits for the classification of retinas compromised or not by AMD. Despite this work presenting good results (AUROC = 0.991), we believe it is very dependent on the mean thickness measures and does not analyze how the local variation of thickness occurs along the RPEDC surface. So, we realize it is possible to propose a new approach for describing RPEDC conditions with local thickness variation analysis. Submitting these features to a classification method allows us to develop a methodology for the detection of AMD in human eyes.

Given the similarity of the generated retinal topographic maps with geological investigation maps like soil analysis and remote sensing [22], we propose using the geostatistical functions of semivariogram and semimadogram as descriptors for characterization of the macular region of the retina.

These geostatistical functions have already been used with success in biomedical contexts such as lung nodule detection [23] and breast cancer classification [24]. In these works, geostatistical functions are applied directly on the intensity values of pixels in regions of the image to differentiate between healthy tissue or tissue compromised by cancer. In [25], these functions are used to automatically detect eyes, differentiating them from other facial features. We can see that these methods well describe the texture of tissue or a topographic map, like proposed in this work.

The main advantage of using geostatistical functions over topographic maps is that they allow a representation of the volume as a whole, not limiting the analysis by individual slices (not being subject to the misalignment of slices problem) and providing a local analysis of thickness variation along the RPEDC in macular region.

In this work, we used the topographic map proposed by [5] but, innovate on not use the topographic map to describe only the thickness of the layers. Otherwise, we try to describe the global texture presented in the topographic map. The texture representation of the topographic map is the central contribution/innovation point of the proposed work.

To describe the macular region texture we propose the use of the semivariogram and semimadogram functions that we believe can describe the layers thickness variations of the total retina, NSR, e RPEDC. Thus, the complete methodology is innovative in itself, as it is the use of geostatistical functions to discriminate against the texture of the topographic map. This is a way to represent the retina globally.

Our approach is innovative in the sense that it uses the analysis of local variation of the thickness as a texture map, based on the assumption that druse formation modifies these measures. This gives us a way to evaluate thickness information with their spatial location building a robust method for AMD detection.

The occurrence of druses interferes in the retinal layers morphology, deforming them which reflects on generated topographic maps. Thus, our method of AMD diagnosis is based on the evaluation of the retinal layers thickness alterations. The topographic maps describe how their thickness is varying along the macular region. So it is an appropriate tool to be used in automatic methods. We developed a way to describe these variations based on the assumption that the texture representation of this maps will be a strong indication of healthy and unhealthy patients.

So, we believe this methodology may be useful to more robustly distinguish impairment cases and let us develop a computer-based method for automatic diagnosis of AMD impairment. Such a system can offer a second opinion, to provide more information to the specialist, supporting their clinical decision. Processing that data through machine learning methods can allow rapid studies, facilitating population screening, remote diagnosis and even early detection of the disease. The problem addressed in this research will be research on a methodology for the automatic diagnosis of AMD by using SD-OCT exams.

## Methods

**Fig. 1:**
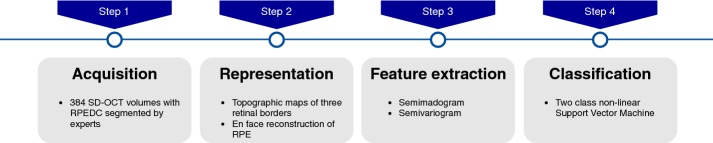
Flow chart of the proposed methodology. This picture shows a view of the necessary stages to perform AMD diagnosis over SD-OCT images

The methodology proposed in this work for the development of a CADx architecture is based on the following stages: (1) image acquisition, (2) image representation, (3) features extraction and (4) classification. The flow chart of the proposed methodology is shown in Fig. 1.

In the first step, the images are acquired from the database provided by [5] which contain precise markings of the borders of the total retina, RPEDC, and neurosensory retina. In the second step, we generate topographic maps and reconstruction *en face* of the SD-OCT volumes. The third step is the central part of the proposed methodology where we extract geostatistical features on the topographic maps obtained from SD-OCT volumes. Finally, in the fourth stage, we submit the extracted features to a classifier method to determine if an SD-OCT volume is whether or not affected by AMD. We detail each step below.

### Image acquisition

The image set used for the validation of the proposed methodology belongs to the database provided by [5]. It is a subset of the AREDS2 [26] database containing 269 eye volumes with AMD and 115 control volumes (eyes considered normal). This data was collected for the purpose of studying quantitative indicators on the presence of AMD in adults.

The main population characteristics of the AREDS2 trials are: Mean age, 74 years; 57% female; 97% white; 7% current smokers; 19% with prior cardiovascular disease; and 44% and 50% taking statin-class cholesterol-lowering drugs and aspirin, respectively. Ocular characteristics include 59% with large bilateral drusen, 32% with advanced AMD in 1 eye and mean visual acuity of 20/32 in eyes without advanced AMD [26].

The SD-OCT examinations were captured at four clinics using SD-OCT scanners manufactured by Bioptigen, Inc (Research Triangle Park, NC). The volumes were acquired in the rectangular region of $6.7 \,{\text {mm}} \times 6.7$ mm centered on the fovea with rapid scan protocol, resulting in volumes of size $1000 \times 512 \times 100$ voxels.

The subset provided by [5] (also used in this work) has subjects aged between 50 and 85 years exhibiting intermediate AMD with druses greater than $125\,\upmu$m in both eyes or large drusen in one eligible eye and advanced AMD in the fellow eye, with no history of vitreoretinal surgery or ophthalmologic disease that might affect acuity in either eye. The control group selection used the same inclusion criteria in the AREDS2 study with the exception that the subject did not present any evidence of the presence of drusen.

**Fig. 2:**
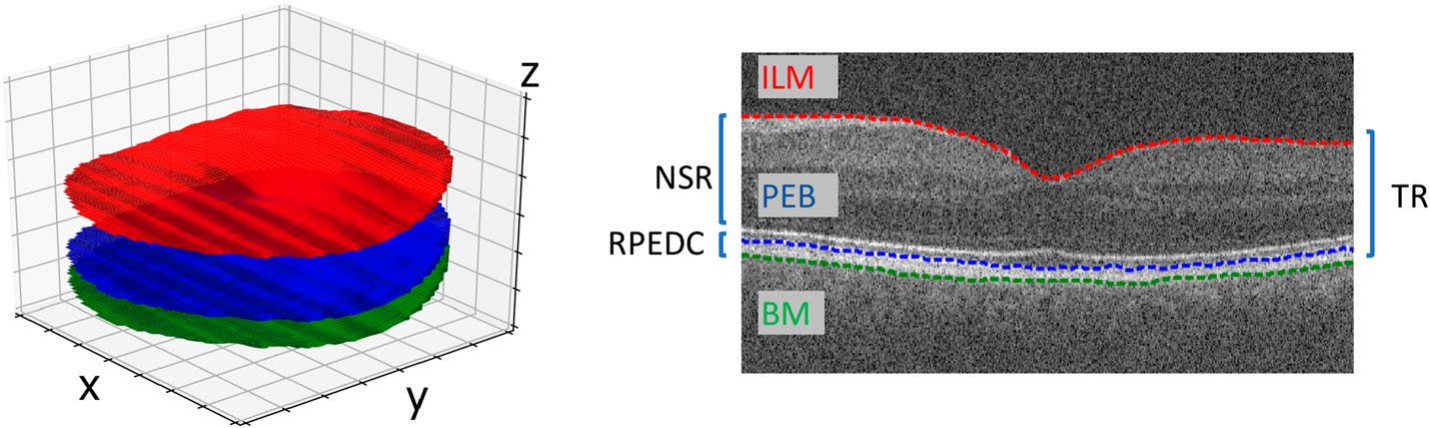
Representation of SD-OCT layer’s boundaries marks. The image on the left represents the surfaces that determine the marking of the borders of the retina divisions. The right image, in turn, is a B-Scan of the same volume extracted from the image base provided by [5] with emphasis on the demarcation of the borders. In both images the borders that delimit the neurosensorial retina (NSR) and the retinal pigmented epithelium and drusen complex (RPEDC) are represented. The NSR is delineared by internal layer membrane (IML, colored red) and the pigmented epithelium border (PEB, colored blue). In turn, the retinal pigmented epithelium and drusen complex (RPEDC) is delineared by PEB and Bruch’s membrane (BM, colored green) including the drusenoids alterations. The total retina (TR) comprehend the whole region between IML and BM

This image set provided segmented retinal layer borders that limit the total retina (inner limiting membrane and Bruch’s membrane) that were segmented altogether with the border of the retinal pigment epithelium. This segmentation was initially performed automatically by the software DOCTRAP, developed by Duke University, and subjected to careful review and adjustments by certified specialists from Duke Advanced Research in Spectral Domain OCT Imaging Laboratory. We present a single B-Scan of an SD-OCT volume in Fig. 2.

After segmentation, the specialists manually marked the center point of the fovea. From this marking, they delimited a cylinder centered at this point as the area of interest of each volume. For tests and validation of this step, we utilized all 384 provided volumes and its layers boundaries marks.

In spite of the existence of automated segmentation techniques, at the moment, we use the segmentation provided with the base due to it being reviewed by specialists. This gives us greater security for validation of the proposed method since segmentation errors may impact its accuracy.

### Image representation

In this step, we generated two-dimensional representations based on topographic maps and reconstruction *en face* for each SD-OCT 3D volume of the image set. Topographic maps represent the thickness of the retina (as well as the neurosensory layer and the RPEDC) for each point on its surface. The reconstruction *en face* generates images that correspond to the reflectance values of the SD-OCT on the border of the RPE. The image set used represents borders reviewed by specialists as surfaces $B_i=(x, y)$, $i\in \{1, 2, 3\}$. These three areas correspond respectively to the i limiting membrane (ILM), border of the pigment epithelium and the border of Bruch’s membrane (BM). These surfaces are used for the representations as detailed below.

#### Generation of topographic maps

From the surfaces defining the borders of the layers, we generated three two-dimensional surfaces $L_i(x, y)$, where $i\in \{1, 2, 3\}$, to represent each SD-OCT 3D volume. The coordinate $z_i = L_i(x, y)$ corresponds to the axial depth of a point located in the coordinates (*x*, *y*) in a substructure of the retina. This is done by summing the amount of voxels existing between the two borders, or simply calculating $L_1 = B_3 - B_1$, $L_2 = B_2 - B_1$ and $L_3 = B_3 - B_2$.

**Fig. 3:**
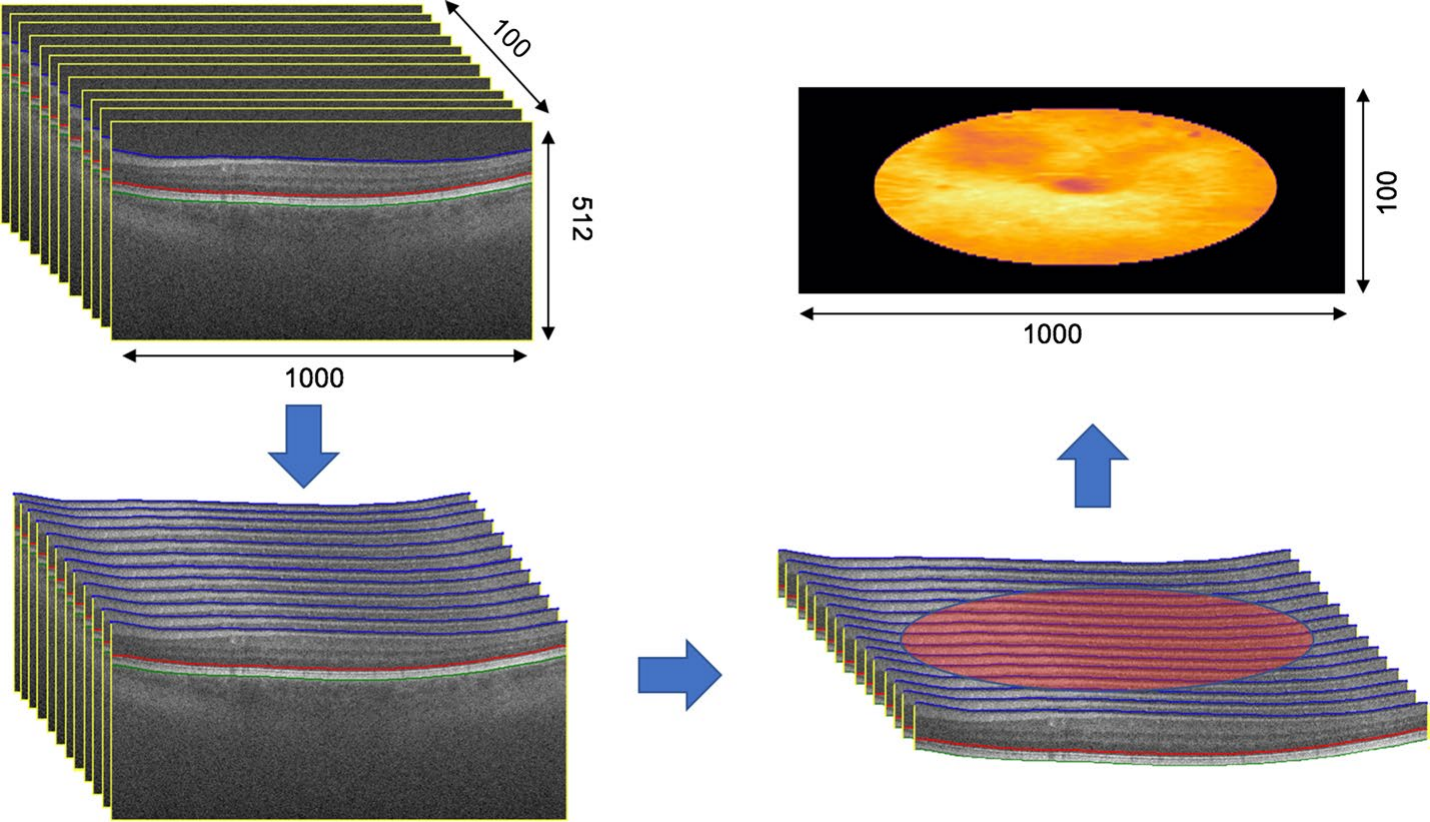
Total retinal topographic map generation from a SD-OCT volume. Complete volume is represented in A. B and C presents successive elimination of unnecessary regions. B also demonstrates the delimitation of a ROI with radius $r = 5$ mm centered in the marking of fovea marked by specialists. Finally, D presents the topographic map that represents the thickness of the retina for each point

The first topographic map generated (surface $L_1$) represents the total retina thickness, corresponding to the region between the border of the internal limiting membrane (ILM) and the border of Bruch’s membrane (BM). The second map (surface $L_2$) uses the neurosensorial retina (NSR), region between the ILM and the border of the pigment epithelium. The last topographic map ($L_3$) corresponds to the retinal pigment epithelium and drusen complex (RPEDC), which is the region between the BM and the border of the RPE. In order to better illustrate, Fig. 3 presents the generation of the topographic map for the surface $L_1$.

#### *En face* reconstruction of RPE

The reconstruction *en face* of the border of the pigment epithelium generates a two-dimensional surface $F_{RPE}$ representing the SD-OCT volume in which the reflectance values existing in the inner border of the pigment epithelium are associated to each pixel. Thus, an image is extracted bearing resemblance to a depiction of an eye fundus. However, each pixel presented corresponds to the voxels located on the border of the RPE, in accordance to:

1 $$\begin{aligned} F_{RPE} = R(L_2,x,y,z) \end{aligned}$$

where, R is the $L_2$ surface reflectance for position *x*, *y* and *z*.

A distance parameter *d* can be specified to take into account 2*D* neighboring voxels located at the maximum distance of *d* up or down. Therefore, the representation *en face* is generated by the average values obtained from this set of voxels. The purpose of this representation by average is to render it more robust in relation to noise.

**Fig. 4:**
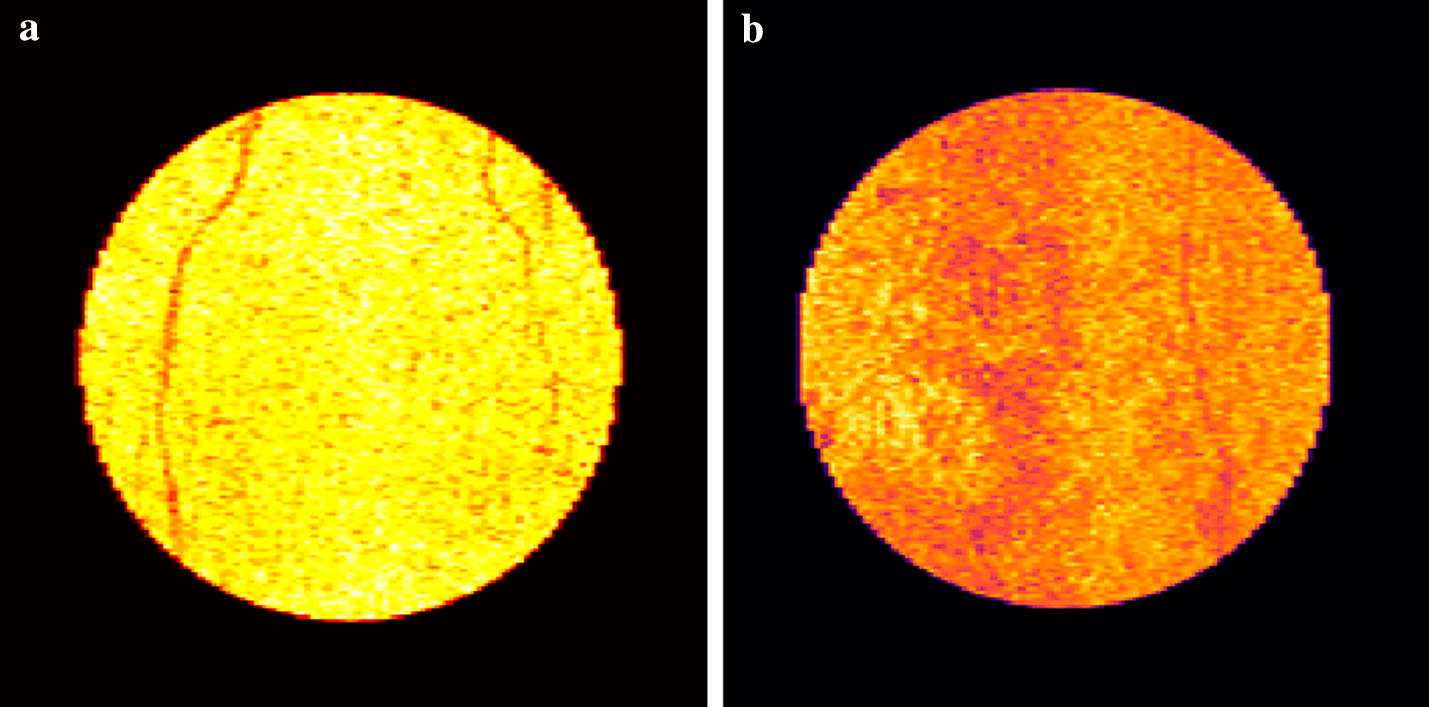
Reconstruction *en face* of the border of the RPE. The figure shows the surface of the pigmented epithelium for a retina of the control group (**a**) and a retina with a diagnosis of AMD (**b**). The clearer values represent points of greater reflectance

Figure 4 presents the reconstruction *en face* of the border of REP for a random volume with AMD and another considered normal. It is possible to visually perceive variations in the texture of the two images, where the darker regions correspond to points of lesser reflectance such as blood vessels and drusen formation.

### Feature extraction

A key concept in geostatistical image analysis is the notion of spatial continuity, which represents the likelihood of a particular pixel value at a determined location given neighboring or regional data values [27]. Thus, it allows the study of the randomness of the sampled data in order to identify possible spatial structures. Events occurring in a particular location tend to have similar behavior in neighboring locations. This characterizes what we call spatial dependence.

The degree of spatial dependence (or variablity of the data) between samples (pixels, in this case) can be measured by semivariance. It analyzes the variance between pairs of data points over a range of distances. Its magnitude is directly proportional to the distance between the points, so the larger the distance, the larger the semivariance value.

#### Semivariogram

The semivariance values as a function of distance between pair of points is called semivariogram and can be written as [28]:

2 $$\begin{aligned} \gamma (h)= \frac{1}{2N(h)} \sum _{(i,j)|h_{ij}=h} (DN_i - DN_j)^2 \end{aligned}$$

where the parameter $h_{i,j}$ called *lag* is the distance between the pairs of points located in *i* and *j* where $DN_i$ and $DN_j$ are respectively the values of pixels *i* and *j*. *N*(*h*) is the number of points separated by *h*.

**Fig. 5:**
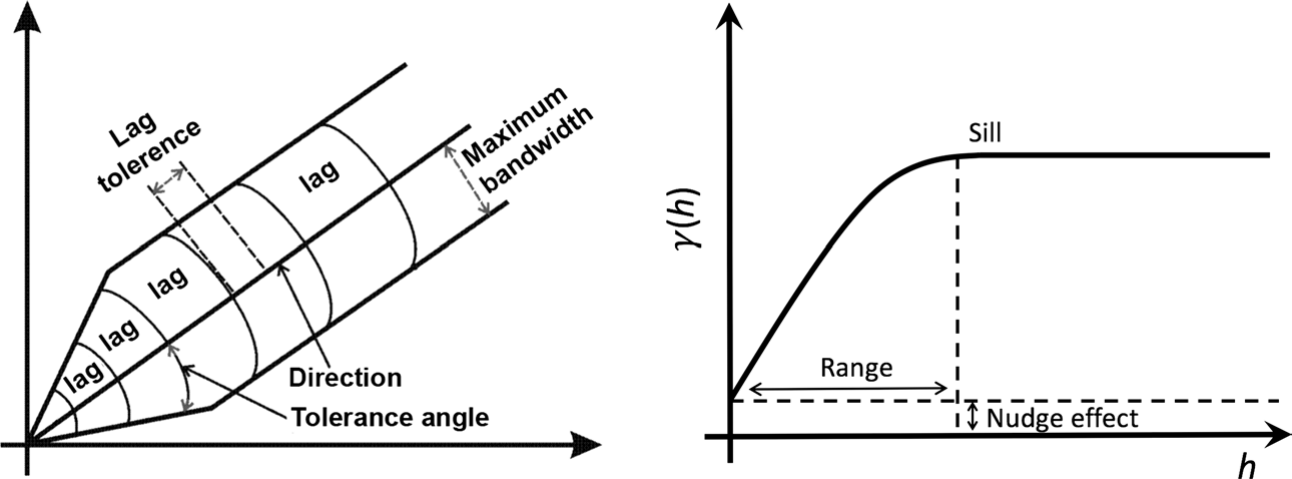
Semivariogram parameters (left) and the characteristic curve (right)

The semivariogram usually displays a characteristic curve (Fig. 5) in which semivariance grows from small lags to large lags. At a given distance the semivariance becomes approximately constant. The plateau where $\gamma$ stabilizes is called the *sill*.

The distance at which the sill is reached is called *range*. At this point, the semivariance is equal to the variance of the data of the sample considered, meaning that there is no relation between the pairs of observations considered at this distance [23]. On the other hand, if the distance *h* is very small, the compared points are very similar to each other, which implies very low values of semivariance for these initial distances, hence the value of the semivariogram will start with very low values $\gamma > 0$. This value, as well as the range and the sill, also represents a characteristic of the semivariogram called the *nugget effect*, or simply *nugget*.

Some parameters are used for the calculation of the semivariogram: the direction, the measurement (or lag distance), the maximum bandwidth, the lag tolerance and the angular tolerance. Setting these values limits which pixels will be considered for the calculation. Figure 5 show an illustration of the boundaries applied by these parameters to the pairs of *pixels*.

#### Semimadograma

Similar to the semivariogram, the semimadogram is another function also used to measure spatial dependence [23]. It is given by the mean of the absolute difference measured in the sample pairs as a function of distance and direction. The function is defined by:

3 $$\begin{aligned} m(h)= \frac{1}{2N(h)} \sum _{i=1}^{N(h)}|x_i - y_i| \end{aligned}$$

where *h* is the *lag*, $x_i$ and $y_i$ are the values of *pixels* of an image separated by *h*. *N*(*h*) is the number of pairs from the distance *h*.

To represent the instances in feature vectors, we calculated semivariogram and semimadogram over the topographic maps of RT($L_1$), NSR($L_2$) and RPEDC($L_3$). These functions were also calculated on the reconstruction *en face* of the border of the RPE. The calculations were performed in four directions ($0^\circ , 45^\circ , 90^\circ$ and $135^\circ$) that are commonly used in computational image processing. The values of *lag* = $h \in \{1, 2, 3, ..., 15\}$, the tolerance for *lag* $\Delta h = \,\pm 3$, tolerance for the direction angle was equal to $10^\circ$ and the maximum bandwidth of 3 *pixels*. The feature vectors are used as inputs for the classification step.

### Classification

To classify between AMD and non-AMD cases, we have used the Support Vector Machines (SVM) [29] which is a supervised learning method for data classification and pattern recognition. Its principle is based on separating data in a sample space from training on a set of previously labeled data. The separation of these data is done through hyperplanes that segment the sample space into partitions, thus separating the instances into distinct classes [30].

SVM is a proven effective method for general classification tasks. In spite of other classifiers that require large amounts of data, such as deep learning [14], SVM is a very robust classifier for tests with a few amount of samples (384 in our case).

For evaluating the performance of the classifier, we used the measures of sensitivity, specificity, accuracy, AUROC and Cohen’s kappa [31]. Sensitivity is defined by $TP/(TP + FN)$, specificity is defined by $TN/(TN + FP)$, and accuracy is defined by $(TP + TN)/(TP + TN + FP + FN)$, where *TN*, is true-negative, *FN* is false-negative, *FP* is false-positive, and *TP* is true-positive.

## Results

To evaluate the performance of the proposed methodology, we run an experiment with the 383 SD-OCT volumes provided by [5]. The missing volume was not used due to a smaller number of B-Scans it contains in comparison with the others (80 B-Scans versus 100 in the other volumes). Testing was performed on a computer with an Intel Core i7 processor with 8 GB of RAM. The implementation was fully developed in the Python 3.5 language in a Debian Linux environment.

The semivariogram and semimadogram for different topographic maps were calculated by generating eight different arrangements of maps/geostatistical functions to be evaluated for the ability to distinguish examination affected by AMD from those considered healthy. The arrangements are: (1) total retina (semivariogram), (2) total retina (semimadogram), (3) neurosensorial retina (semivariogram), (4) neurosensorial retina (semimadogram), (5) RPEDC (semivariogram), (6) RPEDC (semivariogram), (7) RPEDC *en face* (semivariogram) and (8) RPEDC *en face* (semimadogram).

**Fig. 6:**
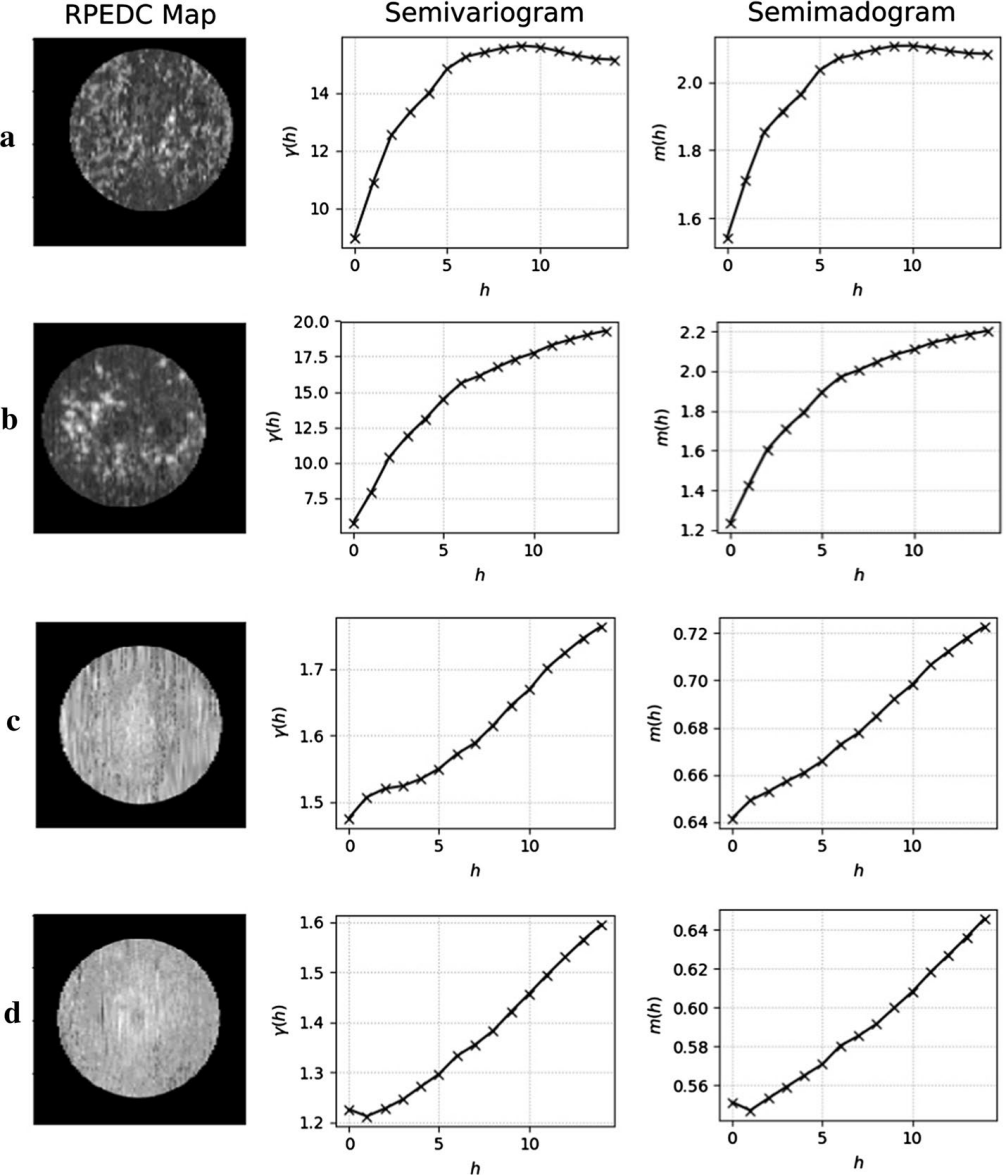
Semivariogram and semimadogram response for RPEDC Maps. Rows A and B correspond to exams afflicted by AMD, while C and D present control volumes. The first image of each row corresponds to the volume’s representation through RPEDC’s topographic map and the last two columns respectively show the semivariogram and semimadogram functions in the $45^{\circ }$ direction

For each arrangement, the geostatistical functions provide values that show local spatial dependency of the chosen maps. In Fig. 6, we show the output given by the geostatistical functions for two cases diagnosed with AMD and two control cases. This data is then submitted to the SVM classifier permitting the realization of tests and validation.

The validation tests were performed using the k-fold technique with repetitions. For training, we divided the data into 5 folds with 100 repetitions, setting a ratio of 80% for training and 20% for tests in each interaction. Using a large number of repetitions increases the reliability of the results given the fact that it decreases the contribution of folds that present overfitting to the overall result. We calculated the mean values, standard deviations for each test (Table 2).

As can be seen in Table 2, we describe that both the semivariogram and semimadogram when calculated on the topographic map of the total retina have low differentiation capacity between the two classes. The average AUROC found for these tests are, respectively, 0.576 and 0.756.

When using the topographic map of the neurosensory retina, we obtained better results in terms of sensitivity, specificity and AUROC. The best results for this map were obtained by the semimadogram function with average sensitivity of 90.0%, specificity mean of 84.3%, accuracy mean of 88.3%, and AUROC mean of 0.950. The kappa index of 0.724 indicates a substantial agreement.

We obtained the best results with topographic maps of the RPEDC, especially with semivariogram function. The average accuracy of $95.2\%$ shows that this model has an excellent diagnostic differentiation capability for AMD, presenting an average AUROC of 0.989.

On the other hand, the semivariogram an semimadogram functions applied on RPE’s reconstruction *en face*, which considered the reflectance of the points in the upper border of this layer, did not demonstrate expressive ability to differentiate the control tests from examinations affected by AMD (average AUROC of 0.505 and 0.778, respectively).

**Fig. 7:**
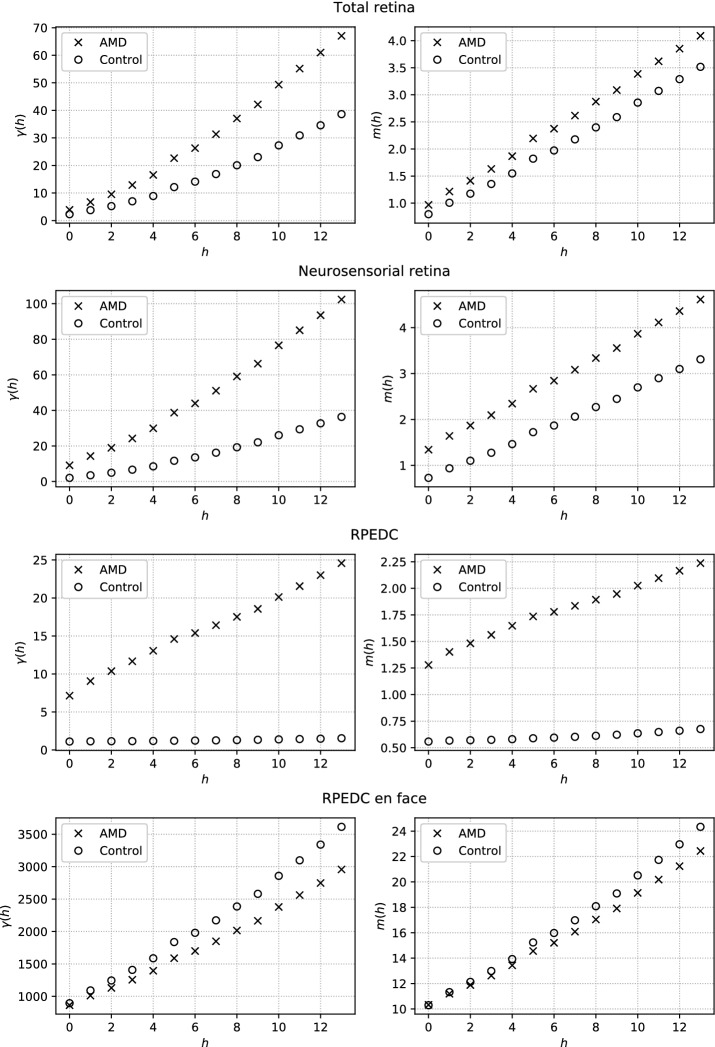
Semivariogram and semimadogram functions plots for $45^\circ$ of the retinal layers. The vertical scale is different for each layer

For a visual comparison, in Fig. 7 we present the semivariograms and semimadograms responses calculated for the topographic maps. These were calculated for direction of $45^\circ$ and a volume with AMD and a control one are directly compared.

**Table 2:** Results of AMD classification based on geostatistical features

Feature	Fold	Sensitivity (%)	Specificity (%)	Accuracy (%)	AUROC	Kappa
TR (SV)	Average	76.0	42.3	65.8	0.576	0.180
	Std	5.7	10.2	4.7	0.071	0.101
	Max acc	84.5	61.1	78.9	0.306	0.439
	Max kappa	84.5	61.1	78.9	0.306	0.439
TR (SM)	Average	91.9	26.0	71.7	0.756	0.204
	Std	5.7	13.9	5.3	0.052	0.123
	Max acc	96.7	50.0	86.8	0.846	0.541
	Max kappa	91.2	63.2	84.2	0.821	0.564
NSR (SV)	average	88.8	65.7	81.9	0.802	0.554
	Std	4.1	9.6	3.8	0.060	0.091
	Max acc	96.5	80.0	92.2	0.861	0.791
	Max kappa	96.5	80.0	92.2	0.861	0.791
NSR (SM)	Average	90.0	84.3	88.3	0.950	0.724
	Std	4.0	7.0	3.2	0.023	0.076
	Max acc	98.2	95.5	97.4	0.993	0.936
	Max kappa	98.0	96.3	97.4	0.996	0.943
*RPEDC (SV)*	*Average*	*94.2*	*97.5*	*95.2*	*0.989*	*0.886*
	Std	3.1	3.2	2.3	0.010	0.054
	Max acc	100.0	100.0	100.0	1.000	1.000
	Max kappa	100.0	100.0	100.0	1.000	1.000
RPEDC (SM)	Average	90.2	91.6	90.5	0.977	0.780
	Std	4.5	7.5	3.5	0.014	0.080
	Max acc	100.0	95.7	98.7	0.999	0.969
	Max kappa	98.0	100.0	98.7	0.992	0.971
RPEDC *en face* (SV)	Average	100.0	0.0	70.2	0.505	0.000
	Std	0.0	0.0	4.7	0.008	0.000
	Max acc	100.0	0.0	84.4	0.542	0.000
	Max kappa	100.0	0.0	71.4	0.500	0.000
RPEDC *en face* (SM)	Average	90.2	41.5	75.5	0.778	0.347
	Std	4.7	10.1	4.3	0.051	0.098
	Max acc	95.1	62.5	88.3	0.844	0.619
	Max kappa	95.1	62.5	88.3	0.844	0.619

The data in italics represents the best values obtained

**Fig. 8:**
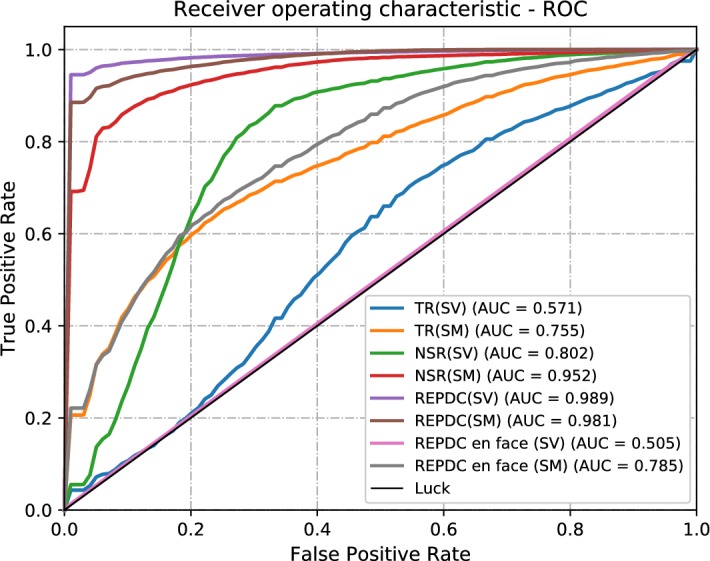
ROC plots of each generated SVM model. The best obtained value was 0.989

We further compare the performance of our best method (semivariogram over RPEDC) to the classical texture descriptors (Haralick) [32, 33] and with the Local Binary Patterns (LBP) [33] to evaluate the performance of our methods in comparison to the commonly used descriptors that were previously mentioned (Fig. 8).

**Fig. 9:**
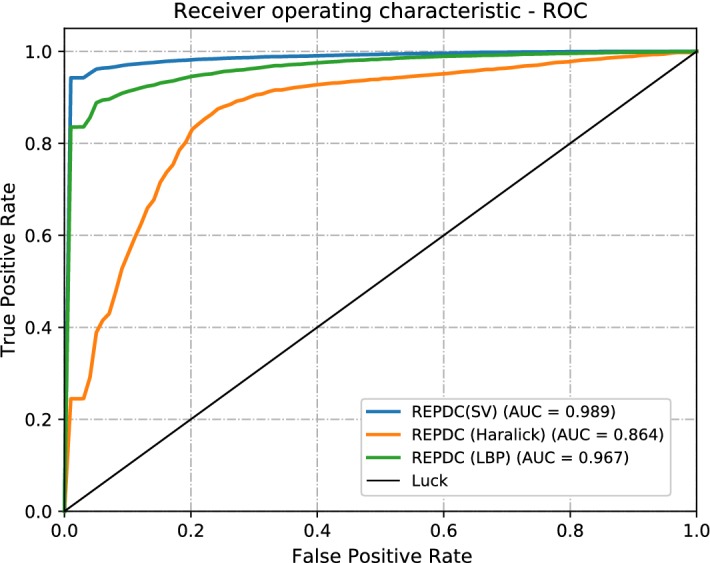
ROC plot for comparison of semivariogram peformance with another methods. The best obtained value was 0.989

The results of these experiments (Table 3) show that the commonly used descriptors possess lower values in comparison to the semivariogram descriptor. Specifically, the LBP and Haralick descriptor methods achieved AUROCs of 0.968 and 0.862 respectively. These values are lower than the best result presented in this work (arrangement 5: AUROC = 0.989). Table 3 presents the comparison between these results and Fig. 9 shows a graphical comparison of the different AUROC values.

**Table 3:** Comparison with others techniques over same data

Test	Sen (%)	Spc (%)	Acc (%)	AUROC	Kappa
Classical texture features	88.7	77.4	85.3	0.862	0.651
Local Binary Patterns	93.0	86.1	90.9	0.968	0.784
Semivariogram (this work)	94.2	97.5	95.2	0.989	0.886

## Discussion

Table 2 describes that both the semivariogram and the semimadogram when calculated on the topographic map of the total retina have low differentiation capacity for AMD detection. This was also demonstrated in [5] by using thickness average maps. Specifically in our work, this result indicates that there is no significant difference in terms of spatial dependence between classes when considering the total retina.

When using the topographic map of the neurosensory retina, the kappa index of 0.724 indicates a substantial agreement. Notwithstanding, these values are still slightly below the results found in the literature. The main difference between the results obtained by the maps of the total retina and the neurosensory retina is that the last map is calculated from the upper border of the RPE, a layer that is directly deformed from the accumulation of drusen.

The best results (semivariogram on the RPEDC) achieved an AUROC of 0.989, a result very close to that achieved by [5]. The value of $kappa = 0.886$ also indicates that despite being validated with an unbalanced sample, the model’s performance possesses a high concordance between classes.

On the other hand, the semivariogram applied on the reconstruction *en face* of the RPE, which solely considered the reflectance of points in the upper border of this layer, did not demonstrate expressive ability to differentiate the control tests of the examinations affected by AMD. Especially the semivariogram, which resulted in an average AUROC of 0.505, which is a result too similar to a random classifier. A slightly better AUROC (0.778) was achieved when using the semimadogram for this reconstruction. However, a lower kappa value (0.347) indicates that the generated model is biased and tends to present a high number of mismatched classifications.

The poorest performance of this last test against the promising results obtained with the topographic map of the RPEDC is due to the fact that the AMD does not cause significant changes in the reflectance of points on the border of the RPE as with the map of thickness. These variations are best observed when evaluating the deformations caused in this layer by the appearance of drusen and possibly choroidal neovascularization. In future work, we can take into consideration not only the border, but also an axial integration of the pixels of the RPE and thus identify whether reflectance changes in this layer are significant for the detection of AMD.

In Fig. 7 we present a visual comparison between the plotting of semivariogram and semimadogram functions relating to various tests performed. Therefrom, we can perceive that the semivariogram function produces very close values for the classes when applied on the map of the total retina and reconstruction *en face* of the RPE. The values, in turn, become well differentiated when applied on the topographic map of RPEDC, congruent with the best generated model.

Considering the results achieved, we verified that the best model generated for the diagnosis of AMD using topographic maps is arrangement 5 (RPEDC/Semivariogram). We noticed that the performance of the proposed model is greater than various works that are based in reflectance values. A comparison with related works is presented in Table 4.

**Table 4:** Comparison of performance of the main methodologies presented in related works

Work	Volumes	Acc	Sen	Spc	AUROC
Liu et al. [4]	457	89.3%	–	–	0.975
Serrano et al. [7]	200	–	96.0%	92.0%	–
Albarrak et al. [8]	140	91.4%	92.4%	90.5%	0.944
Zhang et al. [10]	140	92.06%	91.82%	92.3%	–
Farsiu et al. [5]	384	–	–	–	0.991
Srinivasan et al. [9]	45	–	100%	–	–
Venhuizen et al. [11]	384	–	–	–	0.984
Wang et al. [12]	45	93.3%	–	–	0.995
Sun et al. [13]	45	100%	–	–	–
	678 B-Scans	99.6%	–	–	–
Ravenscroft et al. [15]	75	83.3%	–	–	–
Fang et al. [16]	45	100%	100%	100%	1.00
	54	92.2%	96.9%	95.4%	–
Karri et al. [17]	45	89.0%	–	–	–
Lee et al. [18]	100,000 B-scans	88.98%	85.41%	93.82%	0.938
Kermany et al. [19]	207,130 B-scans	99%	98%	99.2%	0.999
This work	383	95.2%	94.2%	97.5%	0.989

Some works have presented slightly higher accuracy, however they use smaller image sets for validation, and other ones have used individualized B-Scans. They do not perform an overall evaluation of the retina, which makes it difficult to compare the work directly. It is also important to note that the performance information is not presented in some papers.

Among the works that make use of the same SD-OCT volumes base [5, 11] along with the tests that were made with the Haralick and LBP techniques, we show through the results achieved with the semivariogram over RPEDC’s topographic map that the proposed method is robust for the detection of AMD.

With the direct comparison between AUROC values, we can see that the results are really promising. The work of [5] (which presents better AUROC) is based on the mean thickness of the retinal layers, which may vary slightly depending on the AMD stage and it does not consider the local variation of these values. We believe that our method has the advantage of acting on the pattern of the distribution of anatomical changes caused by AMD.

Haralick descriptors also do not make use of a spatial distribution parameter in their analysis. Lastly, although LBP does consider the data’s spatial localization, it was not able to adequately discriminate volumes with compromised retinas, which brings us to consider the use of a some type of pre-processing for this technique.

Also, we intend to investigate this in the future with other databases that are available, or with a base generated in conjunction with a local clinic.

We believe that the proposed method’s results, which is based on the local thickness variation, are promising. Although the AUROC found is slightly smaller than [5], it must be emphasized that the used base is small and there also exists the necessity of testing our method with bases that present cases of early AMD or that contain smaller differences in RPEDC thickness.

## Conclusions

In this article, we proposed a methodology for automated diagnostic of AMD in SD-OCT images using geoestatistical semivariogram and semimadogram functions as descriptors. These descriptors were applied to the topographic map of the total retina as well as sublayers (neurosensory and retinal pigment epithelium) of the macular region together with the reconstruction *en face* of the pigment epithelium surface. The data was submitted to a Suport Vector Machine classifier after the feature extraction.

We exclusively applied geostatistical texture descriptors for the detection of AMD in optical coherence tomography images base. This is done by a texture classification of the topographic map of human retina layers and this method can be used as a disease screening tool or follow-up in studies of new treatments and prevention methods.

Considering that the main objective of this work was to demonstrate that these descriptors are effective for the diagnosis of AMD, we believe that we have achieved this goal with assuredly promising results. The best performed model presented an AUROC of 0.989. The validation of this model demonstrates that the proposed methodology can distinguish with excellent accuracy AMD from unafflicted eyes. Nevertheless, it is advisable to test with a larger database with larger varieties of thickness and even try the method with other pathologies that may affect the retina.
