# Correction to: Semivariogram and Semimadogram functions as descriptors for AMD diagnosis on SD-OCT topographic maps using Support Vector Machine

**DOI:** 10.1186/s12938-018-0598-x

**Published:** 2018-11-08

**Authors:** Alex M. Santos, Anselmo C. Paiva, Adriana P. M. Santos, Steve A. T. Mpinda, Daniel L. Gomes, Aristófanes C. Silva, Geraldo Braz, João Dallyson S. de Almeida, Marcelo Gattas

**Affiliations:** 1 Federal University of Maranhão UFMA, Applied Computing Group - NCA, Av. dos Portugueses, SN, Campus do Bacanga, Bacanga, São Luís, MA 65085-580 Brazil; 2 Instituto Federal de Educação, Ciência e Tecnologia do Maranhão, São José de Ribamar, MA Brazil; 3 Université du Québec à Montréal, Montréal, Canada; 4 Pontifical Catholic University of Rio de Janeiro PUC-Rio, R. São Vicente, 225 Gávea, Rio de Janeiro, RJ 22453-900 Brazil

## Correction to: BioMed Eng OnLine (2018) 17:160 10.1186/s12938-018-0592-3

After publication, it was highlighted that the original publication [1] contained a spelling mistake in the first name of Marcelo Gattas. This was incorrectly captured as Marelo Gattass in the original article which has since been updated.
